# Doravirine-associated resistance mutations in antiretroviral therapy naïve and experienced adults with HIV-1 subtype C infection in Botswana

**DOI:** 10.1016/j.jgar.2022.08.008

**Published:** 2022-12

**Authors:** Ontlametse T. Bareng, Sekgabo Seselamarumo, Kaelo K. Seatla, Wonderful T. Choga, Blessing Bakae, Dorcas Maruapula, Nametso Kelentse, Natasha O. Moraka, Baitshepi Mokaleng, Patrick T. Mokgethi, Tsotlhe R. Ditlhako, Molly Pretorius-Holme, Mpaphi B. Mbulawa, Refeletswe Lebelonyane, Ebi Celestin Bile, Tendani Gaolathe, Roger Shapiro, Joseph M. Makhema, Shahin Lockman, Max Essex, Vlad Novitsky, Sununguko W. Mpoloka, Sikhulile Moyo, Simani Gaseitsiwe

**Affiliations:** a Botswana Harvard AIDS Institute Partnership, Gaborone, Botswana; b Department of Medical Sciences, Faculty of Allied Health Professions, University of Botswana, Gaborone, Botswana; c Department of Biological Sciences, Faculty of Science, University of Botswana, Gaborone, Botswana; d Division of Human Genetics, Department of Pathology, Faculty of Health Sciences, University of Cape Town, Cape Town, South Africa; e Division of Medical Virology, Stellenbosch University, Cape Town, South Africa; f Department of Immunology and Infectious Diseases, Harvard T.H. Chan School of Public Health, Boston, Massachusetts, USA; g Ministry of Health, Republic of Botswana, Gaborone, Botswana; h FHI 360, Department of Clinical Sciences, Durham, North Carolina, USA; i Brigham and Women's Hospital, Boston, Massachusetts, USA

**Keywords:** HIV-1C, Drug resistance mutations, Antiretroviral (ART) experienced, Antiretroviral (ART) naive, Doravirine, Botswana

## Abstract

**Objectives:**

There are limited data on the prevalence of doravirine (DOR)-associated drug resistance mutations in people with HIV (PWH) in Botswana. This cross-sectional, retrospective study aimed to explore the prevalence of DOR-associated resistance mutations among ART-naïve and -experienced PWH in Botswana enrolled in the population-based Botswana Combination Prevention Project (BCPP).

**Methods:**

A total of 6078 HIV-1C *pol* sequences were analysed for DOR-associated resistance mutations using the Stanford HIV drug resistance database, and their levels were predicted according to the Stanford DRM penalty scores and resistance interpretation. Virologic failure was defined as HIV-1 RNA load (VL) >400 copies/mL.

**Results:**

Among 6078 PWH, 5999 (99%) had known ART status, and 4529/5999 (79%) were on ART at time of sampling. The suppression rate among ART-experienced was 4517/4729 (96%). The overall prevalence of any DOR-associated resistance mutations was 181/1473 (12.3% [95% confidence interval {CI}: 10.7–14.1]); by ART status: 42/212 (19.8% [95% CI: 14.7–25.4]) among ART-failing individuals (VL ≥400 copies/mL) and 139/1261 (11.0% [95% CI: 9.3–12.9]) among ART-naïve individuals (*P* < 0.01). Intermediate DOR-associated resistance mutations were observed in 106/1261 (7.8% [95% CI: 6.9–10.1]) in ART-naïve individuals and 29/212 (13.7% [95% CI: 9.4–8.5]) among ART-experienced participants (*P* < 0.01). High-level DOR-associated resistance mutations were observed in 33/1261 (2.6% [95% CI: 1.8–3.7]) among ART-naïve and 13/212 (6.1% [95% CI: 3.6–10.8]) among ART-failing PWH (*P* < 0.01). PWH failing ART with at least one EFV/NVP-associated resistance mutation had high prevalence 13/67 (19.4%) of high-level DOR-associated resistance mutations.

**Conclusion:**

DOR-associated mutations were rare (11.0%) among ART-naive PWH but present in 62.7% of Botswana individuals who failed NNRTI-based ART with at least one EFV/NVP-associated resistance mutation. Testing for HIV drug resistance should underpin the use of DOR in PWH who have taken first-generation NNRTIs.

## Highlights

• Lower DOR-resistance in ART-naïve versus ART-experienced individuals with VF.
• DOR-resistance was higher among individuals failing NNRTI-containing regimens.
• DOR use in NNRTI-experienced population should be based on HIV drug resistance data.

## 1 Introduction

The use of antiretroviral therapy (ART) has had a significant effect on the HIV-1 epidemic, turning it into a chronic manageable disease [1,2]. The available FDA-approved HIV antiretroviral drugs (ARVs) have increased over time and have improved efficacy and toxicity profiles [3,4]. However, the development of HIV drug resistance mutations (DRMs) threatens the success of combination ART (cART) and further limits future HIV treatment options [5,6]. Recently, a rise in the prevalence of HIV DRMs to first-generation ART non-nucleoside reverse transcriptase inhibitor (NNRTI)-containing regimens has been observed [7], [8], [9], [10], which has contributed to the use of a dolutegravir (DTG)-based first-line ART regimen. DTG has proven to have high efficacy and a higher genetic barrier of resistance [11]. In June 2016, the Botswana National ART program adopted DTG-based first-line therapy for all people with HIV (PWH) initiating ART [12]. Prior studies have reported the development of HIV variants harbouring DTG resistance, especially in highly treatment-experienced patients with previous raltegravir exposure, and these patients tend to harbour variants with multidrug resistance mutations [13,14]. In addition, higher DTG-associated virologic failure (VF) was observed among NNRTI-experienced individuals harbouring NNRTI-associated resistance [15]. This highlights the need for new ART post-DTG, which lack cross-resistance with the current ARV regimens.

A newly approved third-generation NNRTI, doravirine (DOR), has been shown to be more potent, with distinct resistance patterns compared to the earlier-generation NNRTIs [16]. DOR has a better safety profile and has a long half-life of about 12–21 h, enabling it to be dosed once a day [17,18]. In vitro studies have shown that DOR is efficacious in the presence of most common reverse transcriptase resistance mutations, K103N and Y181C, found in NNRTI-experienced individuals [16,19]. To extend these in vitro studies, it is important to evaluate whether DOR can maintain its potency in a population with a high prevalence of the K103N and Y181C mutations, such as PWH in Botswana. We investigate the prevalence of DOR-associated resistance mutations in ART-naïve and ART-experienced PWH in Botswana, where NNRTIs were part of first-line ART regimens from 2002 to 2016. These data could guide policy on DOR use in the Botswana ART program and other countries where HIV-1 subtype C predominates and have similar ART experiences.

## 2 Methods and materials

### 2.1 Study population

PWH aged 16–64 years in 30 communities in northern, central and southern parts of Botswana participated in the Botswana Combination Prevention Project (BCPP) between 2013 and 2018 [20]. The BCPP impact evaluation survey enrolled 16 years or older individuals, regardless of HIV or ART status, who were residing in a random sample of approximately 20% of households among 30 communities at study entry (baseline household survey [BHS]) into a longitudinal cohort (annual household survey [AHS]) that was followed for 30 months as described elsewhere [20]. In addition to 15 intervention communities (15 villages that received preventative measures such as three-drug ARV to all people with viral loads of 10 000 copies/mL and/or CD4 cells ≤500 to track HIV incidence), PWH who were receiving ART in local clinics were recruited to provide a one-time blood sample for HIV sequencing and metadata but were excluded on the longitudinal cohort. For this analysis, samples and data from all PWH in both the AHS cohort and from the clinics were used.

### 2.2 Selection of study participants

**Figure 1:**
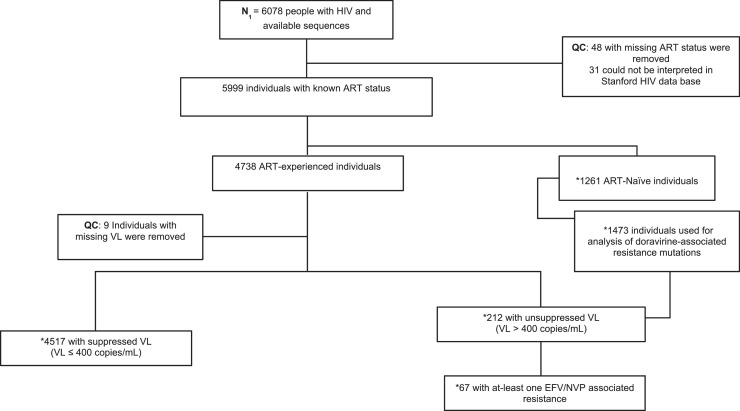
Study schema for the participants used to determine the prevalence of DOR-associated resistance mutations.

In this analysis, we included PWH who were either ART-naïve or ART-experienced, who had HIV-1 viral load (VL) measurement at the first BCPP study visit and had available HIV-1 sequence. HIV-1 VL of participants was quantified using Abbott m2000sp/rt assay (Wiesbaden, Germany) with a range of 40–10 000 000 copies/mL [21]. Among participants who were on ART, some were classified as having VF (HIV-1 VL >400 copies/mL, as per current Botswana's HIV treatment guidelines definition) [22]. Participants with HIV-1 VL ≤400 copies/mL were regarded as virally suppressed (Fig. 1).

### 2.3 Near full-length HIV genotyping

Both HIV-1 proviral DNA sequences and viral RNA sequences were generated by a long-range HIV genotyping protocol described elsewhere [21,23]. The next-generation sequencing (NGS) was performed by the BioPolimers Facility at Harvard Medical School (https://genome.med.harvard.edu/) and through collaboration with PANGEA HIV consortium [21] at the Wellcome Trust Sanger Institute (Cambridge, UK; http:// www.sanger.ac.uk/) with high-sequencing coverage using Illumina platforms MiSeq and HiSeq [21].

### 2.4 HIV-1 subtyping

Generated near full-length HIV-1 sequences were subtyped by online tools REGA version 3 [24] and COMET [25].

### 2.5 Analysis of HIV-1 drug resistance mutations

DOR-associated resistance mutations were identified according to the lists of surveillance drug resistance mutations and major drug resistance mutations (DRMs) in the Stanford University HIV Drug Resistance Database algorithm 9.1 (https://hivdb.stanford.edu/hivdb/by-sequences/). The level of DOR resistance was predicted according to the Stanford HIV DRM penalty scores and resistance interpretation (individuals with low-level, intermediate-level and high-level resistance were considered to have drug resistance). Only intermediate and high-level specific DOR-associated resistance mutations were reported. The list of mutations assessed are shown in Table 1.

**Table 1:** List of DOR-associated mutations assess, their penalty scores and resistance level

Mutations	DOR penalty score	Resistance level
V106M	30	Intermediate
Y188F	30	Intermediate
G190Q	30	Intermediate
L234I	45	Intermediate
V106A	60	High-level resistance
Y188L	60	High-level resistance
G190E	60	High-level resistance
F227C	60	High-level resistance
F227I	60	High-level resistance
F227L	60	High-level resistance
F227V	60	High-level resistance
M230L	60	High-level resistance
Y318F	60	High-level resistance

The prevalence of DOR-associated resistance was estimated in the following groupings: (i) overall; (ii) ART-naïve individuals; (iii) virologic failure (VF) on ART; and (iv) viral suppression (HIV-1 RNA ≤400 copies/mL) on ART. The DOR prevalence was compared between groups (ii) and (iii), as well as between groups (iii) and (iv). The prevalence of specific DOR-associated resistance mutations was estimated within each group and compared among groups. We also assessed the presence of DOR-associated resistance mutations in a subset of participants on NNRTI-based regimens with at least one major efavirenz (EFV)/nevirapine (NVP)-associated resistance mutation (L100I, K101E/P, K103N/S, V106A/M, Y181C/I/V, Y188L/C/H, G190A/S/E and M230L).

### 2.6 Apolipoprotein B mRNA editing enzyme, catalytic polypeptide-like (APOBEC)-induced hypermutations

Guanine-to-adenine transitions (G-to-A) apolipoprotein B mRNA editing enzyme, catalytic polypeptide-like (APOBEC)-induced hypermutations were screened in the viral sequences using the Hypermut program available in Los Alamos National Laboratory HIV Database tools (http://www.hiv.lanl.gov/) [26]. Adjustment for hypermutations was performed [23] using the HIV-1C consensus sequence as a reference. The adjusted hypermutations were accounted for using the cumulative number of mutations across the length of the sequences from the analysed HIV-1 *pol* gene. The adjustment for hypermutations was performed before the drug resistance analysis, as a part of quality control. HIV DRMs identified as hypermutations were not included in the prevalence of DOR resistance in this study.

### 2.7 Statistical analysis

Patient demographics between ART-naïve and individuals on ART experiencing VF were compared using Wilcoxon rank-sum test for continuous variables such as plasma log_10_ HIV-1 VL and χ^2^ test for categorical variables such as sex. The prevalence of mutations was estimated with 95% confidence using the binomial exact method for each group. The prevalence of mutations among ART-naïve group and individuals experiencing VF on ART was compared using comparison of proportions test. The potential association between the prevalence of HIV-DRM and sex, age, EFV use, NVP use, log_10_ HIV-1 VL was assessed by univariate logistic regression (adjusted for clustering by community) in NNRTI-failing individuals with at least one EFV/NVP-associated resistance mutation for detection of DOR-associated resistance mutations. A *P* value <0.05 was considered statistically significant. All the statistical analysis was done using STATA version 14 software.

### 2.8 Ethical considerations

The BCPP study was approved by the Institutional Review Board (IRB) at the U.S. Centers for Disease Control and Prevention and the Botswana IRB (HRDC), and it is registered at ClinicalTrials.gov (NCT01965470). All recruited participants provided written informed consent for participation.

## 3 Results

### 3.1 Participants characteristics

The median age at enrolment of the participants included in this analysis was 34 years (interquartile range [Q1, Q3: 27.1, 42.0]), and study participants were mostly women (66%). Among 6078 participants, 5999 (99%) had known ART status (either ‘on ART’ or ‘ART-naive’). The majority of these participants, 4738 (79%), were on ART, whereas 1261 (21%) were ART-naïve at the time of sampling. Among 4738 participants, a total of 4729 had HIV-1 VL data; 4517 (96%) were virally suppressed (HIV-1 VL ≤400 copies/mL). The median HIV-1 VL among ART-naïve individuals (4.3 [Q1, Q3: 3.5,4.8] log_10_ copies/mL) was similar to the median HIV-1 VL among individuals experiencing VF on ART (4.0; [Q1, Q3: 3.2, 4.7] log_10_ copies/mL; *P* = 0.07; not shown). The study entry demographics of BCPP participants stratified by HIV-1 VL groups are in Table 2: females, older participants (>29 years) and those on ART were most likely to have HIV-1 VL< 400 copies/mL.

**Table 2:** General characteristics of study participants included

	Total *N = 5990 (%),* median (Q1, Q3)	Viral suppression ON ART *n* = 4517, median (Q1, Q3)	Individuals with virologic failure on ART N = 212, median (Q1, Q3)	ART-naïve *n* = 1261, median (Q1, Q3)	*P* values
Sex		*n* = 4516			
Females	4243 (71)	3265 (73)	137 (65)	841 (67)	<0.01
	1746 (29)	1251 (27)	75 (35)	420 (33)	
Males					
Age (y)		*n* = 4516			
<30	1018 (17)	509 (11)	73 (34)	436 (35)	<0.01
30–39	1964 (33)	1459 (32)	74 (35)	431 (34)	
40–49	1767 (29)	1484 (33)	39 (18)	244 (19)	
50+	1240 (21)	1064 (24)	26 (12)	150 (12)	
Geographical region					
North	1490 (25)	1223 (27)	53 (25)	214 (17)	<0.01
South	1745 (29)	1185 (26)	70 (33)	490 (39)	
Central	2755 (46)	2109 (47)	89 (42)	557 (44)	
Year of sampling					
2013–2015	3368 (56)	2471 (55)	97 (46)	800 (63)	<0.01
2016–2018	2622 (44)	2046 (45)	115 (54)	461 (37)	
Median log_10 VL_ copies/ML	1.6 (1.6, 2.6)	1.6 (1.6, 1.6)	4.0 (3.2, 4.7)	4.3 (3.5, 4.8)	<0.01^a^
Known ART regimens (N = 4318)		*n* = 4161	*n* = 157	n/a	<0.01^b^
Non-NNRTI	584 (13)	534 (13)	50 (32)		
EFV		2455 (59)	82 (52)		
NVP	1197 (28)	1172 (28)	25 (16)		

ART, antiretroviral therapy; EFV, efavirenz-based therapy; IQR, 95% confidence intervals; *n*, population size; non-NNRTI, other ART therapy except non-nucleoside reverse transcriptase inhibitors, non-NNRTI-lopinavir, and dolutegravir-containing regimens; NVP, nevirapine-containing regimen, 1672 with missing ART regimen information.

a *P* values obtained using Wilcoxon rank-sum test, while other *P* values were from χ^2^ test.

b *P* values obtained from viral suppression and individuals with virologic failure on ART, numbers in brackets are percentages within columns.

### 3.2 Overall prevalence of DOR-associated resistance, intermediate and high-level resistance levels with specific mutations among ART-naïve and individuals experiencing VF on ART

A total of 1473 participants with viremia were analysed for DOR-associated resistance mutations. Of these, 1261 were ART-naïve, whereas 212 were individuals experiencing VF on ART with HIV-1 VL >400 copies/mL. The overall prevalence of participants with DOR-associated resistance mutations was 181/1473 (12.3% [95% CI: 10.7–14.1]). Higher overall prevalence of DOR-associated resistance mutations was reported among individuals experiencing VF on ART, which was 42/212 (19.8% [95% CI: 14.7–25.4]) compared to 139/1261 (11.0% [95% CI: 9.3–12.9]) in the ART-naïve population (*P* < 0.01). A majority of the participants harboured intermediate DOR-associated resistance mutations with a lower prevalence of 106/1261 (7.8% [95% CI: 6.9–10.1]) in ART-naïve individuals and 29/212 (13.7% [95% CI: 9.4-19.1]) among ART-experienced participants (*P* < 0.01). The prevalence of high-level DOR-associated resistance mutations was 33/1261 (2.6% [95% CI: 1.8–3.7]) among ART-naïve individuals and 13/212 (6.1% [95% CI: 3.6–10.8]) among individuals experiencing VF on ART (*P* < 0.01). Of the two intermediate DOR-associated mutations reported in the study (V106M and Y188F), V106M was the most predominant mutation, 12/1473 (0.8%), and was common among individuals experiencing VF on ART 7/212 (3.3%) (*P* < 0.01). Seven high-level DOR-associated mutations predicted were V106A, Y188L, F227L and M230L, which occurred in 2/1473 (0.14%) participants, F227C and Y318F in 1/1473 (0.07%) each and the G190E 33/1473 (2.2%), which was the most predominant mutation. The prevalence of specific DOR-associated resistance mutations stratified by ART groups are summarized in Table 3.

**Table 3:** Overall prevalence of doravirine-associated resistance, intermediate and high-level resistance levels with specific mutations among ART-naïve and individuals with virologic failure on ART

Resistance levels	Total *N* = 1473 (%)	ART-naïve *N* = 1261 (%)	Individuals with VF on ART *N* = 212 (%)	*P* values
**Overall resistance**	181 (12.3)	139 (11.0)	42 (19.8)	<0.01
**Intermediate**	135 (9.2)	106 (8.4)	29 (13.7)	<0.01
**High-level resistance**	46 (3.1)	33 (2.6)	13 (6.1)	<0.01
**Specific intermediate mutations**				
**V106M**	12 (0.8)	5 (0.4)	7 (3.3)	<0.01
**Y188F**	2 (0.14)	0	2(0.9)	NA
**Specific high-level mutations**				
**V106A**	2 (0.14)	0	2 (0.9)	NA
**Y188L**	2 (0.14)	0	2 (0.9)	NA
**G190E**	33 (2.2)	30 (2.4)	3 (1.4)	0.36
**F227C**	1(0.07)	0	1(0.5)	NA
**F227L**	2 (0.14)	1 (0.08)	1(0.5)	0.131
**M230L**	2(0.14)	0	2 (0.9)	NA
**Y318F**	1(0.07)	0	1 (0.5)	NA

NOTE: *P* values from comparison of proportions of mutations among ART-naïve and individuals with VF on ART. All mutations reported among individuals with VF on ART were from individuals failing NNRTI with at least one major EFV/NVP-associated resistance mutation. Note that none of the individuals had DOR-associated resistance in the absence of EFV/NVP resistance associated mutations.

ART, antiretroviral therapy; NA, not applicable; VF, virologic failure.

### 3.3 Overall prevalence of mutations associated with intermediate and high-level DOR resistance among individuals failing NNRTI-based ART with at least one EFV/NVP-associated resistance mutation

Amongst individuals experiencing VF on ART with detectable HIV-1 VL >400 copies/mL, 67/212 (31.6% [95% CI: 25.4–38.3]) were failing ART with at least one EFV/NVP-associated resistance mutation. A total of 42/67 (62.7% [95% CI: 50.0–74.2]) had a combination of DOR- and EFV/NVP-associated resistance mutations. The overall prevalence of intermediate DOR-associated resistance mutations was 29/67 (43.3% [95% CI: 31.2–56.0]) in this group, whereas the high-level DOR resistance was reported in 13/67 (19.4% [95% CI: 10.8–30.9]). Mutation V106M (7/67:10.4%) was the most predominant intermediate mutation, followed by Y188F. In the high-level resistance group, G190E was the most prevalent at 3/67 (4.5%), followed by equal proportions of V106A and M230L mutations at 2/67 (3.0%), and, lastly, F227C, F227L and Y318F, all occurring at a prevalence of 1/67 (1.4%) each. Individuals without EFV/NVP-associated resistance mutations had no DOR-associated resistance. Among 67 participants with at least one major EFV/NVP-associated resistance mutation, 38 had dual resistance with at least one major NRTI-associated resistance mutation. The prevalence of DOR-associated resistance mutations was higher in this group: 29/38 (76%) overall prevalence, 21 (55%) intermediate and 8 (21.1%) high-level resistance.

### 3.4 Association of DOR-associated resistance mutations among individuals failing NNRTI-based ART with at least one EFV/NVP-associated resistance mutation

In the univariate logistic regression, among NNRTI failing participants with at least one EFV/NVP-associated resistance mutation, sex, age, EFV use, NVP use and median HIV-1 VL log_10_ copies/mL were not associated with DOR-associated resistance mutations in a small population of 67 participants (Table 4).

**Table 4:** Doravirine-associated resistance mutations in participants on NNRTI-based regimens with at least one EFV/NVP-associated mutations

Characteristics	DOR-associated resistance mutations, n (%)		Univariate analysis	
	Present *n* = 24 (%)	Absent *n* = 43 (%)	Odds ratio; 95% CI	*P* values
Sex				
Males	5 (21)	18 (42)	1.0 (ref)	0.07
Females	19 (79)	25 (58)	0.37; 0.13–1.1	
Age				
<30 years	11 (46)	16 (37)	1.0 (ref)	0.47
>30 years	13 (54)	27 (63)	0.70; 0.27–1.8	
EFV in regimen	8 (33)	11 (26)	1.63; 0.38–7.0	0.51
NVP in regimen	4 (17)	9 (21)	0.61; 0.14–2.6	0.51
HIV-1 VL log_10_ copies/mL, median (IQR)	4.2 (3.5–4.6)	3.5 (3.2–4.7)	1.38; 0.84–2.3	0.21

NOTE: ART regimen information was not available for 35 participants.

ART, antiretroviral therapy; EFV, efavirenz; CI, confidence intervals; DOR, doravirine; IQR, interquartile range; NNRTI, non-nucleoside reverse transcriptase inhibitors; NVP, nevirapine; *n*, population size; VL, viral load.

### 3.5 Overall prevalence of mutations associated with intermediate and high-level DOR resistance among individuals with viral suppression and virologic failure (VF) on ART

We also compared the prevalence of HIV-DRMs associated with DOR resistance among individuals with viral suppression on ART (HIV-1 VL ≤400 copies/mL) compared to individuals experiencing VF on ART. The overall prevalence of mutations associated with DOR resistance was similar among individuals with viral suppression on ART, 910/4517 (20.1% [95% CI: 19.0–21.3]), compared to 42/212 (19.8% [95% CI: 14.7–25.8]) among those experiencing VF on ART (*P* = 0.92). The prevalence of intermediate DOR resistance was 735/4517 (16.3% [95% CI: 15.2–17.4]) among individuals with viral suppression on ART vs 29/212 (13.7% [95% CI:9.4–19.1]) among individuals with VF on ART (*P* = 0.32) and high-level DOR resistance was 175/4517 (3.9% [95% CI: 3.3–4.5]) and 13/212 (6.1% [95% CI: 3.3–10.3]) among individuals with viral suppression and those experiencing VF on ART, respectively (*P* = 0.11), were not statistically different in the ART-experienced population. Mutations reported in the two groups were G190E (161/4729:3.4%), V106M (21/4729:0.4%), F227L (7/4729: 0.15%), Y188L (6/4729: 0.13%), Y318F (5/4729: 0.11%), V106A and M230L were 4/4729 (0.08%) each and F227C (4/4729: 0.04%). The prevalence of DOR-associated mutations is stratified by individuals with viral suppression and VF on ART in Supplementary Table S1.

Of all the 1091 participants with DOR-associated resistance, 230 (21.1% [95% CI: 18.7–23.6]) are likely to have specific DOR-associated resistance mutations and 861 (78.9% [95% CI: 76.4–81.3]) with nonspecific DOR-associated resistance mutations. Among 861 with nonspecific DOR-associated resistance mutations, 850 (98.7% [95% CI: 97.7–99.4]) and 11 (1.3% [95% CI: 0.6–2.3]) had intermediate and high-level DOR-associated resistance, respectively (*P* < 0.01).

## 4 Discussion

To the best of our knowledge, this is the first study to use a large data set with a good representation of the Botswana population to assess the prevalence of mutations associated with DOR resistance among PWH. The reported overall prevalence of DOR-associated resistance was 12.3% higher than 1.62% predicted in the Polish study [27]; however, this prevalence was lower compared to 56.4% [28] and 62% [29] reported in other studies. The difference in the prevalence of DOR-associated resistance across studies may be attributed to the use of different genotyping thresholds to detect DOR-associated resistance mutations, different HIV subtypes, different HIV treatment guidelines in the different studies, and different HIV Stanford algorithm versions used in the analysis.

A significantly lower overall prevalence of DOR-associated resistance was reported among ART-naïve compared to individuals experiencing VF on ART (*P* < 0.01), confirming the opportunity for the use of DOR-containing regimens among PWH who are ART-naïve in Botswana. However, the prevalence of DOR-associated resistance among ART-naïve individuals (11.0%: 139/1261) in this study was statistically higher than the 2.9% prevalence of other NNRTI (first-generation)-associated resistance mutations previously reported in the BCPP study [21] (31/1069, *P* < 0.01). Some studies reported higher resistance to other NNRTI-containing regimens when compared to DOR. A study from Greece, Italy and France reported a lower prevalence of DOR-associated resistance (1.4%) compared to first-generation NNRTI-associated resistance (EFV and NVP; 4.3%), with no association between HIV-1 subtype and presence of DOR-associated resistance mutations [30]. In the HIV-1C settings, the lower prevalence of DOR-associated resistance mutations was reported at 4.7% compared to 9.4% NVP/EFV-associated resistance mutations among ART-naïve population [31].

Our study reports the prevalence of ‘archived’ DOR-associated resistance mutations of 20.1% among individuals with viral suppression on ART, similarly to those with VF on ART. Although the predominant prevalence (62.7%) of DOR-associated resistance mutations was among participants failing NNRTI-based therapy in our findings, this was lower when compared to 84.8% reported in a South African study [31] but higher compared to 42% reported in European studies [32,33]. The prevalence of mutations associated with high-level DOR resistance was low among NNRTI-experienced patients in non-B vs. B HIV-1 subtypes in Italy, with higher prevalence in HIV-1 subtype C; furthermore, the presence of mutation Y188L was associated with HIV-1C subtype [33]. Our results together with other studies support the notion that some DOR-associated resistance mutations are HIV-1 subtype-specific, e.g., intermediate DOR-associated resistance mutation V106M. There is evidence that individuals on DOR harbour distinct resistance profiles of HIV-DRMs compared to other NNRTI regimens [30,34]. Furthermore, there is still a need to understand the mechanisms of DOR-associated resistance, especially among individuals who harbour DOR-associated resistance without specific DOR-associated resistance mutations. The presence of DOR resistance in individuals without specific DOR-associated resistance mutations was observed in phenotypic studies [19,34]; this shows that there could be other mutations that play a part in DOR resistance that are yet to be described.

The high-level DOR-associated resistance mutations G190E, F227L and Y318F all showed a similar prevalence among the three groups: ART-naive, individuals with VF, and individuals on ART with viral suppression. The similar prevalence of these mutations across the three groups highlights the importance of gaining a better understanding of their mechanisms, particularly in ART-naïve and ART-suppressed individuals. It was difficult to determine if the presence of these mutations in ART-naïve individuals was due to undisclosed ART use in the BCPP cohort [35] or to transmitted DRM circulating in the Botswana population [9]. A study conducted in 23/24 individuals with baseline first-generation NNRTI-associated resistance mutations reveals that switching to DOR/lamivudine/tenofovir disoproxil fumarate (DOR/3TC/TDF) leads to viral suppression within 48 weeks of follow-up [36]. These findings suggest that some of the first-generation NNRTI resistance mutations do not have an effect on DOR activity.

EFV has been associated with an increased risk of developing high-level DOR-associated resistance mutations [33], but our findings are discordant with these reports. Although we report no association of EFV use with the presence of DOR-associated resistance, the cohort used had a relatively low number of individuals who were failing NNRTI with at least one EFV/NVP-associated resistance mutation experiencing VF; therefore, our study was not powered to reveal the effect of EFV/NVP-associated resistance in the development of DOR-associated resistance mutations. Nonetheless, our results still highlight the importance of genotyping before initiation of a DOR-containing regimen among individuals with previous NNRTI exposure.

Because our study had a small number of participants on DTG-based ART, our findings cannot reveal whether DOR can be used as an alternative drug for DTG-failing participants with multi-resistance mutations. As a result, future studies should investigate DOR-associated resistance mutations in DTG-failing participants with multi-drug resistance.

In conclusion, the prevalence of intermediate and high-level DOR-associated resistance mutations was low among ART-naïve and ART-failing individuals, with the ART-failing group showing a statistically higher prevalence compared to the ART-naïve group. Specifically, a higher prevalence was observed among PWH with EFV/NVP-associated resistance experiencing VF. Our results support the use of DOR among ART-naïve patients in Botswana; however, we highly recommend HIV drug resistance testing among NNRTI-failing individuals before DOR-based regimen initiation.

## Declaration of Competing Interest

None declared.

## Appendix Supplementary materials

Image, application 1
