# Supplementary material for: Doravirine-associated resistance mutations in antiretroviral therapy naïve and experienced adults with HIV-1 subtype C infection in Botswana
Source: J Glob Antimicrob Resist. 2022 Dec;31:128–34. doi: 10.1016/j.jgar.2022.08.008 (PMC9750894; doi:10.1016/j.jgar.2022.08.008)
Supplement: Supplementary file 1 [file mmc1.docx]

| Resistance levels | Total  N= 4729 (%) | Individuals with Viral suppression on ART  N= 4517 (%) | Individuals with Virologic failure on ART  N=212 (%) | p-values |
| --- | --- | --- | --- | --- |
| Overall resistance | 952 (20.1) | 910 (20.1) | 42 (19.8) | 0.92 |
| Intermediate | 764 (16.2) | 735 (16.3) | 29 (13.7) | 0.32 |
| High-level resistance | 188 (4.0) | 175 (3.9) | 13 (6.1) | 0.11 |
| Specific intermediate mutation  V106M  Y188F | 21 (0.4)  4 (0.09) | 14 (0.3)  2 (0.04) | 7 (3.3)  2(0.9) | <0.01  0.73 |
| Specific High-level mutations  V106A  Y188L  G190E  F227C  F227L  M230L  Y318F | 4 (0.08)  6 (0.13)  161(3.4)  2 (0.04)  7(0.15)  4 (0.08)  5 (0.11) | 2 (0.04)  4 (0.09)  158 (3.5)  1 (0.02)  6 (0.13)  2 (0.04)  4 (0.09) | 2 (0.9)  2 (0.9)  3 (1.4)  1(0.5)  1(0.5)  2 (0.9)  1 (0.5) | <0.01  <0.01  0.1  <0.01  0.17  <0.01  0.08 |

*P-values calculated using comparison of proportion test among individuals with viral suppression and virologic failure on ART.*

Supplementary table 1. Overall prevalence of mutations associated with intermediate and high-level DOR resistance among individuals with viral suppression and virologic failure (VF) on ART
